# Detection of antiviral drug resistance in patients with congenital cytomegalovirus infection using long-read sequencing: a retrospective observational study

**DOI:** 10.1186/s12879-022-07537-6

**Published:** 2022-06-22

**Authors:** Yuka Torii, Kazuhiro Horiba, Jun-ichi Kawada, Kazunori Haruta, Makoto Yamaguchi, Takako Suzuki, Hideko Uryu, Naoyuki Kashiwa, Keiji Goishi, Tomoo Ogi, Yoshinori Ito

**Affiliations:** 1 Department of Pediatrics, Nagoya University Graduate School of Medicine, 65 Tsurumai-cho, Showa-ku, 466-8550 Nagoya, Japan; 2 Department of Genetics, Research Institute of Environmental Medicine Nagoya University, Furo-cho, Chikusa-ku, 464-8601 Nagoya, Japan; 3 Department of Human Genetics and Molecular Biology, Nagoya University Graduate School of Medicine, 65 Tsurumai-cho, Showa-ku, 466-8550 Nagoya, Japan; 4 Department of Pediatrics, National Center for Global Health and Medicine, 1-21-1 Toyama Shinjuku-ku, Tokyo, Japan; 5 Department of Pediatrics and Child Health, Nihon University School of Medicine, 30-1 Oyaguchi, Kami-cho, Itabashi-ku, 173-8610 Tokyo, Japan

**Keywords:** Cytomegalovirus, Ganciclovir, Valganciclovir, Congenital cytomegalovirus infection, Drug resistance, Long-read sequencing

## Abstract

**Background:**

Congenital human cytomegalovirus (cCMV) infection can cause sensorineural hearing loss and neurodevelopmental disabilities in children. Ganciclovir and valganciclovir (GCV/VGCV) improve long-term audiologic and neurodevelopmental outcomes for patients with cCMV infection; however, antiviral drug resistance has been documented in some cases. Long-read sequencing can be used for the detection of drug resistance mutations. The objective of this study was to develop full-length analysis of *UL97* and *UL54*, target genes with mutations that confer GCV/VGCV resistance using long-read sequencing, and investigate drug resistance mutation in patients with cCMV infection.

**Methods:**

Drug resistance mutation analysis was retrospectively performed in 11 patients with cCMV infection treated with GCV/VGCV. *UL97* and *UL54* genes were amplified using blood DNA. The amplicons were sequenced using a long-read sequencer and aligned with the reference gene. Single nucleotide variants were detected and replaced with the reference sequence. The replaced sequence was submitted to a mutation resistance analyzer, which is an open platform for drug resistance mutations.

**Results:**

Two drug resistance mutations (*UL54* V823A and *UL97* A594V) were found in one patient. Both mutations emerged after 6 months of therapy, where viral load increased. Mutation rates subsided after cessation of GCV/VGCV treatment.

**Conclusions:**

Antiviral drug resistance can emerge in patients with cCMV receiving long-term therapy. Full-length analysis of *UL97* and *UL54* via long-read sequencing enabled the rapid and comprehensive detection of drug resistance mutations.

## Background

Human cytomegalovirus (HCMV) is the most common pathogen of congenital infection. Although the incidence varies by race or ethnicity, congenital HCMV (cCMV) infection occurs in approximately one in every 100 to 1000 births [1]. Most infants with cCMV are asymptomatic; however, approximately 10–15% of cCMV cases show physical symptoms [2, 3]. Common findings of symptomatic cCMV include petechiae, jaundice, hepatomegaly, splenomegaly, microcephaly, and other neurological signs. Thrombocytopenia, transaminitis, direct hyperbilirubinemia, chorioretinitis, and neuroimaging abnormalities are indicative of central nervous system involvement, and sensorineural hearing loss can be found on examination [4]. Ganciclovir (GCV) and its oral prodrug, valganciclovir (VGCV), are antiviral agents used in the treatment of symptomatic cCMV in infants. Antiviral treatment initiated within 1 month of life improved neurodevelopmental and hearing outcomes [5]. Furthermore, the 6-month protocol improved hearing and neurodevelopment at the long-term assessment (at 24 months) compared to the 6-week protocol [6].

CMV antiviral drug resistance has been primarily reported in patients with immunosuppression, such as transplant recipients or those with AIDS [7]. Recently, antiviral drug resistance has also been reported in several cases of cCMV receiving GCV/VGCV treatment [8]. Mutations in the viral thymidine kinase gene (*UL97*) and the DNA polymerase gene (*UL54*) confer resistance to GCV/VGCV [9]. The current gold standard for genotypic detection of antiviral drug resistance is Sanger sequencing of PCR-amplified *UL97* and *UL54* gene segments [10]. Although major mutations have been detected in the *UL97* gene [11], several mutations have been reported in *UL97* and *UL54* [9]. Comprehensive antiviral mutation detection with Sanger sequencing is labor-intensive because multiple PCR amplicons are needed to sequence the full-length of *UL54* and *UL97*. Nanopores and long-read sequencing can provide rapid, near real-time sequencing. In this study, full-length antiviral gene mutation analysis was conducted to detect GNC resistance in patients with cCMV infection using nanopore sequencing.

## Methods

### Study design

The objective of this study was to develop full-length analysis of UL97 and UL54, target genes with mutations that confer GCV/VGCV resistance using long-read sequencing. Then, drug resistance mutation in patients with cCMV infection was investigated retrospectively.

### Subjects

This study utilized clinical specimens submitted to the Division of Pediatrics, Nagoya University, between April 2015 and March 2020. Whole blood samples were collected for CMV viral load measurements from patients with cCMV infection who underwent GCV/VGCV therapy. The diagnosis of congenital CMV infection is confirmed by detection of the virus in body fluids within the first 3 weeks of life [12]. GCV/VGCV therapy was indicated for patients with symptomatic cCMV infection based on the following findings: small for gestational age, microcephaly, petechiae, jaundice, hepatosplenomegaly, purpura, intracranial calcification, periventricular cyst or ventriculomegaly, sensorineural hearing loss, or retinitis. Ventriculomegaly was confirmed during fetal ultrasound if the width of the atrium of the lateral ventricle was greater than 10 mm, and then by a pediatric neurologist based on MRI after birth. The duration of the therapy was 6 weeks in the former study period and 6 months in the latter period. The study period was extended in several patients with persistent viremia by the physician’s discretion. The dose of the therapy was 6 mg/kg/dose twice daily for intravenous GCV and 16 mg/kg/dose twice daily for oral VGCV, respectively. The therapy was suspended when the neutrophil count decreased to < 500/µl. The dosage was reduced to half when the neutrophil count became < 1,000/µl after resuming GCV/VGCV or due to any other reasons such as elevated transaminase. Antiviral drug resistance mutation analysis was retrospectively performed in 11 patients with cCMV infection treated with GCV/VGCV. Patients were divided into two groups according to the presence or absence of CMV in whole blood at 6 weeks after the initiation of antiviral therapy (resolution of viremia, n = 4; persistent viremia, n = 7). The assay was performed using the sample collected before therapy in all patients and the sample in which CMV was detected before terminating therapy in the persistent viremia group.

### Validation sample

The cerebrospinal fluid sample used for validation of the antiviral mutation assay was collected from a post-hematopoietic stem cell transplant patient with antiviral mutation (M460V), which had been identified using a conventional method.

### DNA extraction and long-range PCR

DNA was extracted from 200 µL whole blood or cerebrospinal fluid using a QIAamp DNA Blood Mini Kit (Qiagen, Hilden, Germany). DNA was extracted from 140 µL urine using a QIAamp Viral RNA mini kit (Qiagen, Hilden, Germany). Viral loads were measured via real-time PCR using Quantstudio 3 (Applied Biosystems, Foster City, CA), in a total volume of 25 µL composed of 5 µL DNA, 12.5 µL Taqman Fast Advanced Mix (Applied Biosystems), 0.05 µL each of 50 µM sense and antisense primers, 0.025 µL of 100 µM probe, and 7.125 µL of nuclease-free water [13]. The limit of detection of the assay is 100 IU/ml. Residual DNA samples were used for the long-range PCR. The *UL54* and *UL97* genes were amplified as described previously with slight modification [10]. Each 50 µL reaction mixture contained 25 µL LongAmp Hot Start Taq 2× master mix, 400 nM of each primer set (*UL54* primer set; forward; 5’-AGTCCACGCCGCCTCATCTC-3’, reverse; 5’- TCGTAAGCTGTCAGCCTCTCAC-3’, *UL97* primer set; forward 5’- GCAATCCCCGTCACGCCTCTG-3’, reverse; 5’-AACCGTCACGTTCCGCGTCC), 3% dimethyl sulfoxide, and 20 µL DNA as a template. Reactions were run in an Eppendorf Mastercycler (Eppendorf, Hamburg, Germany), using the following cycling parameters: 94 °C for 30 s, 30 cycles at 94 °C for 30 s and 65 °C for 4 min 15 s, and a final extension for 10 min at 65 °C. PCR products (50 µL) were electrophoresed on a 1% agarose gel and stained with Midori Green Advance (NIPPON Genetics, Tokyo, Japan). Bands of target size were cut out from the gel and eluted using NucleoSpin Gel and PCR Clean-Up (Macherey-Nagel, Düren, Germany). The purified PCR products (*UL54* and *UL97*) were pooled per patient for library preparation.

### Nanopore library preparation and sequencing

Nanopore library preparation was performed according to the manufacturer’s instructions for a Ligation Sequencing Kit (SQK-LSK109) (Oxford Nanopore Technologies, Oxford, UK) and a Native Barcoding Expansion 1–12 (EXP-NBD104) (Oxford Nanopore Technologies). Sequencing was performed on a PromethION platform (Oxford Nanopore Technologies) using R9.4.1 flow cells. The library was loaded onto the flow cell according to the manufacturer’s instructions. MinKNOW version 20.06.9 (Oxford Nanopore Technologies) was used to collect and demultiplex raw sequencing data, and Guppy version 4.0.11 (Oxford Nanopore Technologies) was used for base calling raw data after completion of sequencing runs.

### Data analysis

The sequence data output as fastq files was further processed for mutation detection. Each sequenced read was aligned to the reference gene *UL54* (human herpesvirus 5 strain Merlin, NC_006273.2, 78,194 to 81,922, complement) and *UL97* (human herpesvirus 5 strain Merlin, NC_006273.2, 141,798 to 143,921) using Minimap2 [14]. Aligned reads were converted to BAM files and sorted using Samtools [15]. Lofreq is a fast and sensitive variant caller for inferring single nucleotide variants (SNVs) and indels from next-generation sequencing data [16]. Reference gene sequences (UL54.fasta and UL97.fasta) were replaced with SNVs and indels extracted using Lofreq (mutation.fasta).

A mutation resistance analyzer (MRA) is an open platform for antiviral drug mutations published by the University of Ulm [17]. The list of gene mutations can be obtained by uploading mutation.fasta to the MRA platform. The proportion of responsible SNVs for antiviral drug resistance was calculated as the percentage of mutation reads per total reads using the Integrative Genomics Viewer [18].

## Results

### Assay validation

**Fig. 1:**
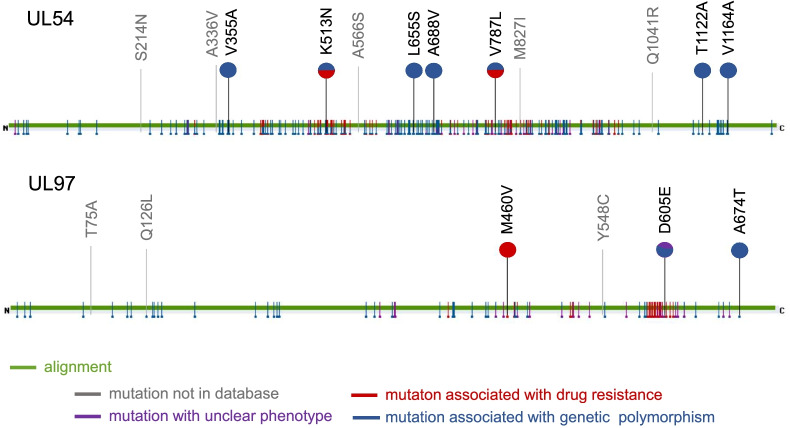
Output images obtained by uploading mutation.fastas to the mutation resistance analyzer (MRA) platform for *UL54* and *UL97*. Reference.fastas for UL54 and UL97 were replaced with SNVs and indels extracted by Lofreq, respectively (UL54 mutation.fasta and UL97 mutation.fasta). The green line indicates that the range of input data is aligned. Each fasta covers the full length of each gene. Any detected mutation is indicated by a colored letter suggesting each phenotype. The pie chart shows the percentage of references reporting each phenotype

To validate our drug resistance mutation assay, the DNA obtained from the cerebrospinal fluid of a patient with a known antiviral mutation (UL97 M460V) [19] was tested. A total of 652,501 and 57,359 reads were aligned to the *UL97* and *UL54* genes, respectively. In addition to M460V in *UL97*, K513N and V787L in *UL54* were detected (Fig. 1). The mutation rates were 68% (428,708/6226,029 reads) in M460V, 74% (39,503/53,339 reads) in K513N, and 48% (24,500/50,634 reads) in V787L (Table 1). To estimate the minimal requirement reads for data analysis, sequence data were randomly sampled to 1000 and 30 reads. The mutation was detected in the sample with 30 reads in a similar proportion to full reads.

**Table 1:** Treatment duration, side effects, and sequelae of cCMV patients with GCV/VGCV

ID	CMV viral load (IU/mL)		Treatment duration	Reduction or suspension of therapy weeks after GCV/VGCV	Side effect	Sensorineural hearing loss		Neuro-development†
	Before GCV/VGCV	6 weeks after GCV/VGCV				at birth	last visit	at 2 years
1	51,761	< LoD*	6 weeks	None	None	None	None	No delay
2	64,649	< LoD	6 months	Suspension (4 W, 6–11 W)	Elevated transaminases	Both ears	No change	Mild
3	88,674	< LoD	6 months	None	None	Right ear	No change	No delay
4	150,000	< LoD	6 weeks	None	None	None	None	Mild
5	87,688	5,099	3 months	None	None	None	None	No delay
6	10,994	7,535	4 months	Suspension (3 W, 9–11 W) Reduction (4–8 W)	Neutropenia	None	None	Mild
7	116,600	31,975	6 months	Suspension (9–10, 12–17 W) reduction (4–8 W)	Neutropenia	None	None	Mild
8	271,309	280,000	3 months	None	None	None	None	Mild
9	18,445	1250	6 weeks	None	None	Both ears	No change	Mild
10	N.A^§^	2149	6 weeks	None	None	Both ears	No change	Mild
11	167,250	199,125	10 months	Suspension (2, 6–7, 29–32 W)	Neutropenia, Elevated transaminases	Right ear	No change	Moderate

*CMV* cytomegalovirus, *GCV* ganciclovir, *VGCV* valganciclovir; * < LoD (IU/): lower than limit of detection; ^§^*N.A.* not available

^†^Neurodevelopment level was defined according to the developmental quotient (DQ) as follows: no delay, > 75, mild 50–75, moderate

### Drug resistance mutation assay in patients with cCMV infection

The clinical manifestations and disease course are summarized in Tables 2 and 3. Therapy was suspended or reduced for four infants because of side effects. Of those patients, worsening of retinal lesions was seen after the second suspension in one infant (patient 11). These lesions subsequently became quiescent. No worsening of clinical signs was seen in the remaining three patients. Five sensorineural hearing loss patients were confirmed by auditory brainstem response before VGCV treatment.

**Table 2:** Assessment of minimal requirement reads for antiviral mutation detection

Gene	*UL54*		*UL97*
Mutation	V787L	K513N	M460V
Gene position	79,564	80,384	143,175
Reference	C	C	A
Full Reads	Total count: 50,643	Total count: 53,339	Total count: 626,029
	A: 696 (1%)	**A: 39,503 (74%)**	A: 185,419 (30%)
	C: 24,055 (47%)	C: 10,983 (21%)	C: 6005 (1%)
	**G: 24,500 (48%)**	G: 2560 (5%)	**G: 428,708 (68%)**
	T: 1392 (3%)	T: 293 (1%)	T: 5897 (1%)
	DEL: 5075	DEL: 2333	DEL: 18,162
	INS: 1278	INS: 1922	INS: 11,573
1000 Reads	Total count: 805	Total count: 833	Total count: 948
	A: 12 (1% )	**A: 596 (72%)**	A: 291 (31%)
	C: 393 (49%)	C: 198 (24%)	C: 7 (1%)
	**G: 382 (47%)**	G: 37 (4%)	**G: 642 (68%)**
	T: 18 (2%)	T: 2 (0%)	T: 8 (1%)
	DEL: 76	DEL: 48	DEL: 21
	INS: 24	INS: 27	INS: 17
30 Reads	Total count: 23	Total count: 24	Total count: 27
	A: 0	** A: 17 (71%)**	A: 10 (37%)
	C: 14 (61%)	C: 5 (21%)	C: 0
	**G: 9 (39%)**	G: 2 (8%)	**G: 17 (63%)**
	T: 0	T: 0	T: 0
	DEL: 2	DEL: 1	DEL: 0
	INS: 0	INS: 2	INS: 2

Single nucleotide variant (SNV) mutation detectable by mutation resistance analyzer (bold)

**Table 3:** Clinical and laboratory imaging findings of congenital cCMV patients with GCV/VGCV

ID	Clinical symptom at birth	Laboratory findings	Brain image findings	Reticulochorioretinitis
1	None	None	Ventriculomegaly	−
2	Purpura	Thrombocytopenia elevated transaminase	Ventriculomegaly, calcification	−
3	None	None	Ventriculomegaly	−
4	None	None	Ventriculomegaly	−
5	None	None	Normal	−
6	Premature birth, gastric rupture	Thrombocytopenia	Ventriculomegaly	−
7	Purpura	Thrombocytopenia	Ventriculomegaly	−
8	Premature birth, ascites, purpura	Thrombocytopenia	Ventriculomegaly	−
9	None	None	Normal	−
10	None	None	Normal	−
11	Premature birth Hepatosplenomegaly	Thrombocytopenia elevated transamynase	Ventriculomegaly, calcification Cerebellar hypoplasia	+

**Fig. 2:**
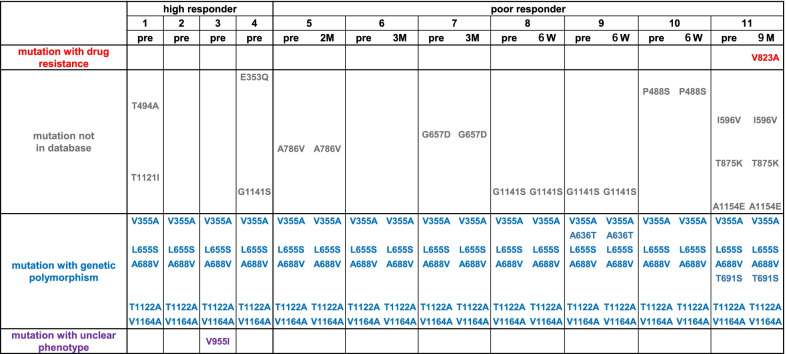
*UL54* mutations in 11 patients with congenital cytomegalovirus (cCMV) infection. The *UL54* mutations detected in 11 patients with cCMV infection were summarized. Each mutation was listed in the order of phenotypes (mutation with drug resistance, mutation not in database, mutation with genetic polymorphism, and mutation with unclear phenotype). *HR* high responder, *PR* poor responder

**Fig. 3:**
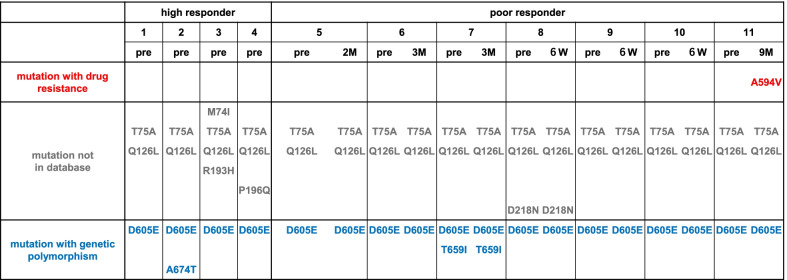
*UL97* mutations in 11 patients with congenital cytomegalovirus (cCMV) infection. The *UL97* mutations detected in 11 patients with cCMV infection were summarized. Each mutation was listed in the order of phenotypes (mutation with drug resistance, mutation not in database, mutation with genetic polymorphism, and mutation with unclear phenotype). *HR* high responder, *PR* poor responder

No antiviral mutation was detected in the sample collected before GCV/VGCV therapy in either group. Two drug resistance mutations (UL54 V823A and UL97 A594V) were found in patient 11, collected at 9 months of therapy in the persistent viremia group (Figs. 2 and 3). No antiviral mutations were detected in other patients in the persistent viremia group.

**Fig. 4:**
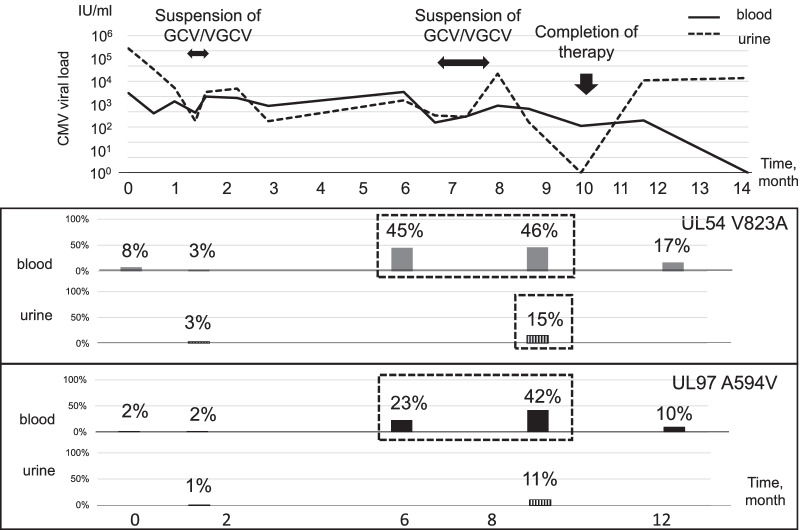
Cytomegalovirus (CMV) viral load and single nucleotide variant (SNV) proportions over time in Pt 11. The CMV load (line graph, bold line: blood, dashed line: urine) and SNV proportion (bar graph; closed bar: blood, hatched bar: urine) of patient 11 are shown over time. Antiviral mutations were detected by Lofreq at the time points in the dashed square

The SNV proportions were further analyzed in a time series (Fig. 4) in patient 11. Both mutation SNVs could be detected at 6 months of therapy in blood when the viral load increased. Mutation SNVs were also detected in urine, but in smaller proportion as compared to those in blood. The V823A mutation was present in a small proportion (8%) at pretreatment, although Lofreq was not significant. In patient 11, there were no clinical signs such as recurrence of retinal lesions or thrombocytopenia around the period in which antiviral resistance emerged. Given that the antiviral mutation had not been investigated at that time, GCV/VGCV treatment continued. Viral load was partially suppressed after 7 months of therapy. VGCV treatment was suspended once; however, it was resumed due to a rebounding viral load and increasing transaminases. The treatment was completed after 10 months of therapy. Viral load rebounded, but clinical symptoms did not worsen. After 12 months, the virus was still detected, though mutation SNV rates subsided after cessation of GCV/VGCV treatment. Urine samples were also analyzed for antiviral mutations, while no such mutations were detected 6 weeks after treatment. After 9 months, mutation rates were slightly increased; however, these rates were smaller than that of the blood sample.

## Discussion

Sanger sequencing is the most frequently used method in antiviral gene mutation assays. As next-generation sequencing (NGS) has become common in clinical settings, an attempt to detect antiviral drug mutations using NGS has been reported [10, 20–23]. Nanopore sequencers can read as long as 100 kbps and are suitable for sequencing the full-length of *UL54* (approximately 4 kbps) and *UL97* (about 2 kbps) genes. As each read covers the full length of the gene, mutations can be detected by a minimum of 30 reads. Minimized input data can reduce the computational load and shorten the time required for data processing.

Data processing can become a bottleneck for NGS assays for clinical researchers who are not familiar with bioinformatics. However, the NGS analysis platform can be used without a bioinformatics background, although there is a limitation in that the user cannot handle the data freely. BugSeq is an online bioinformatics platform for automated microbiology sequencing analysis using nanopore reads [24]. Bugseq can also be utilized in CMV antiviral drug resistance genotyping [20]. In this study, a single set of fastq files was run for Bugseq, and the result matched our results (data not shown). The mutation resistance analyzer is also helpful for collating SNVs with drug resistance mutation [17]. Clinical researchers can utilize such platforms in antiviral research.

The proportion of antiviral mutations can be calculated as the percentage of SNV reads per total reads using NGS [21]. In this study, the SNV proportion was observed over time in patient 11. The percentage of each mutation subsided after cessation of GCV/VGCV treatment in this patient. Repopulation of the wild type after cessation of GCV/VGCV therapy has been reported previously [25]. Sahoo et al. reported the ability to detect an antiviral drug resistance lower than 20% using the NGS method [10]. Although the detection limit of proportion was not set in this study, A594V was detected by Lofreq with a 15% mutation rate in the urine sample of patient 11. Urine samples were also assayed in this study. Interestingly, mutation rates were lower than those in blood samples, although the dynamics of the mutation rates were synchronized with that of blood. This suggests that multiple CMV strains were localized in organs at various rates in the infected individual.

We detected *UL54* mutations in the validation sample (K513N, V787L) and patient 11 (V823A). For the validation sample, the patient received foscarnet after M460V in *UL97* was detected. K513N confers GCV and cidofovir resistance [26], and V787L confers cidofovir and foscarnet resistance [27]. For patient 11, the dynamics of the *UL54* V823A mutation synchronized with *UL97* A594V. V823A has been reported to confer cidofovir and GCV resistance [28]. Most GCV-resistant CMV strains have mutations in *UL97* [11]; however, specific gene mutations in *UL54* may occur, or in combination with *UL97* [7]. The *UL54* mutation in combination with the *UL97* mutation increases the level of resistance [29]. Therefore, *UL97* and *UL54* should also be investigated when the patient is suspected of having antiviral drug resistance using GCV/VGCV.

Several reasons for why drug resistance emerged in patient 11 but no in other infants in the virus persistent group were considered. First, the high viral load before GCV/VGCV therapy in patient 11. This patient might have had a more severe damage in the central nervous system. Retinal abnormalities also suggested an extensive range of viral infection. In contrast, patient 8 had a high viral load before therapy, but antiviral resistance did not emerge. The relatively short period of therapy (3 months) may be one reason. A treatment period longer than 3 months appears to be a risk factor for antiviral drug resistance [30] in immunocompromised patients. Recently, antiviral drug resistance has been reported in patients with cCMV infection [8, 31–35]. It was observed that GCV resistance emerged after 3 months in patients with cCMV infection [8]. A 6-month GCV/VGCV treatment is the recommendation for cCMV therapy [6]; therefore, careful CMV blood load monitoring is needed. Second, an impaired host immune function might be related to persistent viremia. Although the immune function was not fully investigated in patient 11, it was unlikely that the infants had congenital immune dysfunction because the viral load in blood subsided at 14 months of age, 4 months after the cessation of antiviral therapy. The CMV blood load of all patients in this study subsided at approximately 12 months of age. This suggests maturity of the host’s immune system. Third, insufficient blood GCV/VGCV concentrations could not be excluded because of lacking blood GCV/VGCV level measurements. Patient 11 had full-dose VGCV around the period in which the antiviral resistant mutation emerged. In this patient, time-course analysis showed that the mutation emerged after 6 months of therapy. Despite the GCV resistant mutation, it seems VGCV was partially effective because an increase in viral load and transaminases was observed. This may be because the proportion of mutant SNVs did not overwhelm the wild type.

Prolonged detection of CMV in blood could be a risk factor for sensorineural hearing loss [36] in patients with cCMV infection; however, it is sometimes difficult to continue GCV/VGCV therapy because of side effects such as neutropenia. Therefore, when the CMV blood load increases during GCV/VGCV therapy, it is important to consider whether this increase is affected by the ineffective blood GCV/VGCV level or antiviral drug resistance. It is beneficial to monitor CMV viral blood load levels before, during, and after treatment because an increasing viral load during therapy may provide an indication for antiviral resistance, and therefore, an ineffective antiviral therapy. An increasing viral load after reduction or cessation of antiviral therapy because of adverse effects such as antiviral associated neutropenia may provide indication for resuming therapy. Our study is limited by the small sample size of infants with congenital CMV infection. In addition, although nanopore sequencing provides fast long-read sequencing in a compact and portable format and initial low costs, it involves higher base calling error rates when compared to standard next generation sequencing, such as the Illumina platform. Further study could be necessary to better characterize the application of this technology for CMV resistance testing.

In conclusion, an antiviral gene mutation assay was performed using nanopore sequencing. Antiviral drug resistance can emerge in patients with cCMV during long-term GCV/VGCV therapy.

## Data Availability

The datasets analyzed during the current study are available from the. corresponding author Y.I on reasonable request.
